# First Evidence of a Hybrid of *Leishmania (Viannia) braziliensis/L*. *(V*.*) peruviana* DNA Detected from the Phlebotomine Sand Fly *Lutzomyia tejadai* in Peru

**DOI:** 10.1371/journal.pntd.0004336

**Published:** 2016-01-06

**Authors:** Hirotomo Kato, Abraham G. Cáceres, Yoshihisa Hashiguchi

**Affiliations:** 1 Laboratory of Parasitology, Department of Disease Control, Graduate School of Veterinary Medicine, Hokkaido University, Sapporo, Japan; 2 Sección de Entomología, Instituto de Medicina Tropical “Daniel A. Carrión” y Departamento Académico de Microbiología Médica, Facultad de Medicina Humana, Universidad Nacional Mayor de San Marcos, Lima, Peru; 3 Laboratorio de Entomología, Instituto Nacional de Salud, Lima, Peru; 4 Centro de Biomedicina, Facultad de Medicina, Universidad Central del Ecuador, Quito, Ecuador; 5 Prometeo, Secretaría Nacional de Educacion Superior, Ciencia, Tecnologia e Innovacion (SENESCYT), Quito, Ecuador; 6 Department of Parasitology, Kochi Medical School, Kochi University, Kochi, Japan; Fundaçao Oswaldo Cruz, BRAZIL

## Abstract

The natural infection of sand flies by *Leishmania* was examined in the Department of Huanuco of Peru, where cutaneous leishmaniasis caused by a hybrid of *Leishmania (Viannia) braziliensis/L*. *(V*.*) peruviana* is endemic. A total of 2,997 female sand flies were captured by CDC light traps and Shannon traps, of which 2,931 and 66 flies were identified as *Lutzomyia tejadai* and *Lu fischeri*, respectively. Using crude DNA extracted from individual sand flies as a template, *Leishmania* DNA was detected from one *Lu*. *tejadai*. The parasite species was identified as a hybrid of *L*. *(V*.*) braziliensis/L*. *(V*.*) peruviana* on the basis of cytochrome *b* and mannose phosphate isomerase gene analyses. The result suggested that *Lu*. *tejadai* is responsible for the transmission of the hybrid *Leishmania* circulating in this area.

## Author Summary

Leishmaniasis is a protozoan disease caused by members of the genus *Leishmania*, which are distributed worldwide, especially in tropical and subtropical areas. More than 20 species of *Leishmania* are described as causative agents of human leishmaniases and clinical features are largely associated with the infective species. The parasites are transmitted by bites of female sand flies of the genus *Phlebotomus* in the Old World and *Lutzomyia* in the New World. Approximately 800 sand fly species have been recorded; of these, fewer than 10% have been confirmed as vectors of *Leishmania* and only a restricted number of species transmit specific *Leishmania* species. Therefore, sand fly surveillance is important for predicting risk and spread of leishmaniasis in endemic and surrounding areas. Cutaneous leishmaniasis caused by a hybrid of *L*. *(Viannia) braziliensis/L*. *(V*.*) peruviana* have been reported in the Department of Huanuco of Peru; however, the vector species has not been identified. In the present study, approximately 3,000 sand flies were examined, and the sand fly infected by the hybrid *Leishmania* strain was successfully identified.

## Introduction

New World *Leishmanias* are transmitted by phlebotomine sand flies of the genus *Lutzomyia*, and around 480 species have been recorded; in Peru, 149 species have been registered [1], and some of which have been implicated as potential vectors of human *Leishmanias* [2–8]. In Peru, cutaneous leishmaniasis (CL) and mucocutaneous leishmaniasis (MCL) are endemic, and three *Leishmania* species have been identified as predominant causative agents: *Leishmania (Viannia) braziliensis* mainly in the tropical rainforest, *L*. *(V*.*) peruviana* mainly in the Andean highland areas, and *L*. *(V*.*) guyanensis* in the northern and central rainforest regions [9–11]. In addition, distribution of *Leishmania (Leishmania) mexicana*, *L*. *(L*.*) amazonensi*s, *L*. *(V*.*) lainsoni*, *L*, *(V*.*) shawi*, and a hybrid of *L*. *(V*.*) braziliensis*/*L*. *(V*.*) peruviana* were reported [9–12]. Concerning sand flies, prevalent species have been extensively researched, especially in Andean areas [2–7]; however, the vector species responsible for transmission of *Leishmania* have yet to be fully elucidated in most areas because of low infection rates in sand fly populations.

Since 1995, CL cases caused by a hybrid of *L*. *(V*.*) braziliensis/L*. *(V*.*) peruviana* have been reported in the eastern inter-Andean valley of Huanuco province in the Department of Huanuco [12], and the hybrid was suggested to increase disease severity when compared to *L*. *(V*.*) braziliensis* and *L*. *(V*.*) peruviana* using an animal model [13]. Extensive sand fly surveillance revealed prevalent sand fly species and *Lutzomyia (Lu*.*) tejadai* was identified as a dominant species in endemic areas of Huanuco; however, vector species of hybrid *Leishmania* parasite have not been determined to date. In our previous study, a method of mass-screening sand fly vectors for *Leishmania* infections was established and it has become a powerful tool for sand fly research [6,14,15]. In the present study, using the molecular mass-screening method, sand flies from the Department of Huanuco, where CL caused by hybrid *L*. *(V*.*) braziliensis/L*. *(V*.*) peruviana* is endemic, were examined for natural *Leishmania* infections.

## Materials and Methods

### Sand fly collection

Sand flies were collected with CDC light traps set inside houses and Shannon traps outside and around houses at 19 localities in Department of Huanuco (Table 1). CDC light traps were operated throughout the night from 18:00–06:00 and Shannon traps from 18:00–20:00 each night. The sand flies were morphologically identified based on measurements of wing veins, the ratio of length of palpus to length of antenna and the color of the thorax [16], and then fixed in 70% ethanol.

**Table 1:** Sand fly collection in Department of Huanuco.

Province	District	Locality	Sand flies	
			Species	Numbers
**Ambo**	Ambo	Ambo	*Lu*. *tejadai*	292
			*Lu*. *fischeri*	22
	Conchamarca	Ñausilla	*Lu*. *tejadai*	9
	Conchamarca	Sancarragra	*Lu*. *tejadai*	8
	San Rafael	Camahuayin	*Lu*. *tejadai*	344
	Tamay Kichwa	Quicacan	*Lu*. *tejadai*	20
**Huanuco**	Amarilis	Colpa Alta	*Lu*. *tejadai*	17
	Amarilis	Pacan	*Lu*. *tejadai*	266
	Amarilis	San Luis	*Lu*. *tejadai*	21
	Chinchao	Acomayo	*Lu*. *tejadai*	18
	Chinchao	Chayana	*Lu*. *tejadai*	12
	Chinchao	Maray Pampa	*Lu*. *tejadai*	32
	Churubamba	Chinobamba	*Lu*. *tejadai*	569
			*Lu*. *fischeri*	28
	Churubamba	Paca Pucro	*Lu*. *tejadai*	74
	Churubamba	Quechualoma	*Lu*. *tejadai*	117
			*Lu*. *fischeri*	1
	Huanuco	Cabrito Pampa	*Lu*. *tejadai*	59
	Huanuco	Pucuchinche	*Lu*. *tejadai*	43
	Quisqui	Coso Tingo	*Lu*. *tejadai*	714
			*Lu*. *fischeri*	10
	Quisqui	Higueras	*Lu*. *tejadai*	208
	Quisqui	Huancapallac	*Lu*. *tejadai*	108
			*Lu*. *fischeri*	5

### DNA extraction

Ethanol-fixed sand flies were placed individually in each well of 96-well plates and lysed in 50 μl of DNA extraction buffer [150 mM NaCl, 10 mM Tris-HCl (pH 8.0), 10 mM EDTA and 0.1% sodium dodecyl sulfate (SDS)] in the presence of proteinase K (200 μg/ml). The samples were incubated at 37°C overnight and heated for 5 min at 95°C. Each 0.5-μl portion was directly used as a template for mass-screening PCR.

### Detection and identification of *Leishmania* species

Infection of *Leishmania* parasites within sand flies was detected by mass-screening PCR as described previously [6,14]. Briefly, PCR amplification was performed with *Leishmania* minicircle kinetoplast DNA-specific primers (L.MC-1S; 5’-CTRGGGGTTGGTGTAAAATAG-3’ and L.MC-1R; 5’-TWTGAACGGGRTTTCTG-3’) using Ampdirect Plus reagent (Shimadzu Biotech). The PCR products were analyzed on a 2% agarose gel.

*Leishmania* species were identified by *Leishmania* cytochrome *b* (*cyt* b) gene sequence analysis. *Leishmania cyt* b gene fragments were amplified by PCR with a pair of specific primers (L.cyt-S; 5'-GGTGTAGGTTTTAGTYTAGG-3' and L.cyt-R; 5'-CTACAATAAACAAATCATAATATRCAATT-3') using Ampdirect Plus reagent (Shimadzu Biotech, Tsukuba, Japan), and the products were directly cloned into the plasmid using a pGEM-T Easy Vector System (Promega, Madison, WI). The sequence of the insert was determined by the dideoxy chain termination method using a BigDye Terminator v3.1 Cycle Sequencing Kit (Applied Biosystems, Foster City, CA).

### Differentiation between *L*. *(V*.*) braziliensis* and *L*. *(V*.*) peruviana*

Differentiation between *L*. *(V*.*) braziliensis* and *L*. *(V*.*) peruviana* was performed by PCR-RFLP analysis of the mannose phosphate isomerase (MPI) gene. A pair of primers for PCR was designed based on the MPI gene sequences of *L*. *(V*.*) braziliensis* and *L*. *(V*.*) peruviana*. The primer sequences were 5’-GCTCTTCCTGTCGGACAGCGAGC-3’ (MPI-S) and 5’-TCACTCTCGAAGGGAGTTCG-3’ (MPI-R). PCR was carried out in a volume of 15 μl using the primers (0.4 μM each), Ampdirect Plus reagent (Shimadzu Biotech), and *Taq* polymerase (Nova*Taq* Hot Start DNA Polymerase; Novagen, Darmstadt, Germany). After an initial denaturation at 95°C for 10 min, PCR amplification was performed with 35 cycles of denaturation (95°C, 1 min), annealing (55°C, 1 min) and polymerization (72°C, 1 min), followed by a final extension at 72°C for 10 min. Each PCR product was digested with the restriction enzyme, *Ava*II (Takara Bio) and analyzed by 3% agarose gel electrophoresis. Separately, PCR products were purified and the nucleotide sequences were directly determined.

### Phylogenetic analysis

The *cyt* b gene sequences were aligned with CLUSTAL W software [17] and examined using the MEGA program (Molecular Evolutionary Genetics Analysis) version 5.2 using the Kimura two-parameter [18]. Phylogenetic trees were constructed by the neighbor-joining method with the distance algorithms available in the MEGA package. Bootstrap values were determined with 1,000 replicates of the datasets. The datasets for phylogenetic analyses consisted of *cyt* b gene sequences from *L*. *(L*.*) infantum* (GenBank accession number: AB095958), *L*. *(L*.*) donovani* (AB095957), *L*. *(L*.*) major* (AB095961), *L*. *(L*.*) tropica* (AB095960), *L*. *(L*.*) amazonensis* (AB095964), *L*. *(L*.*) mexicana* (AB095963), *L*. *(V*.*) panamensis* (AB095968), *L*. *(V*.*) guyanensis* (AB095969), *L*. *(V*.*) braziliensis* (AB095966), *L*. *(V*.*) peruviana* (AB433282), *L*. *(V*.*) lainsoni* (AB433280), *L*. *(V*.*) naiffi* (AB433279) and *L*. *(V*.*) shawi* (AB433281).

## Results

**Fig 1:**
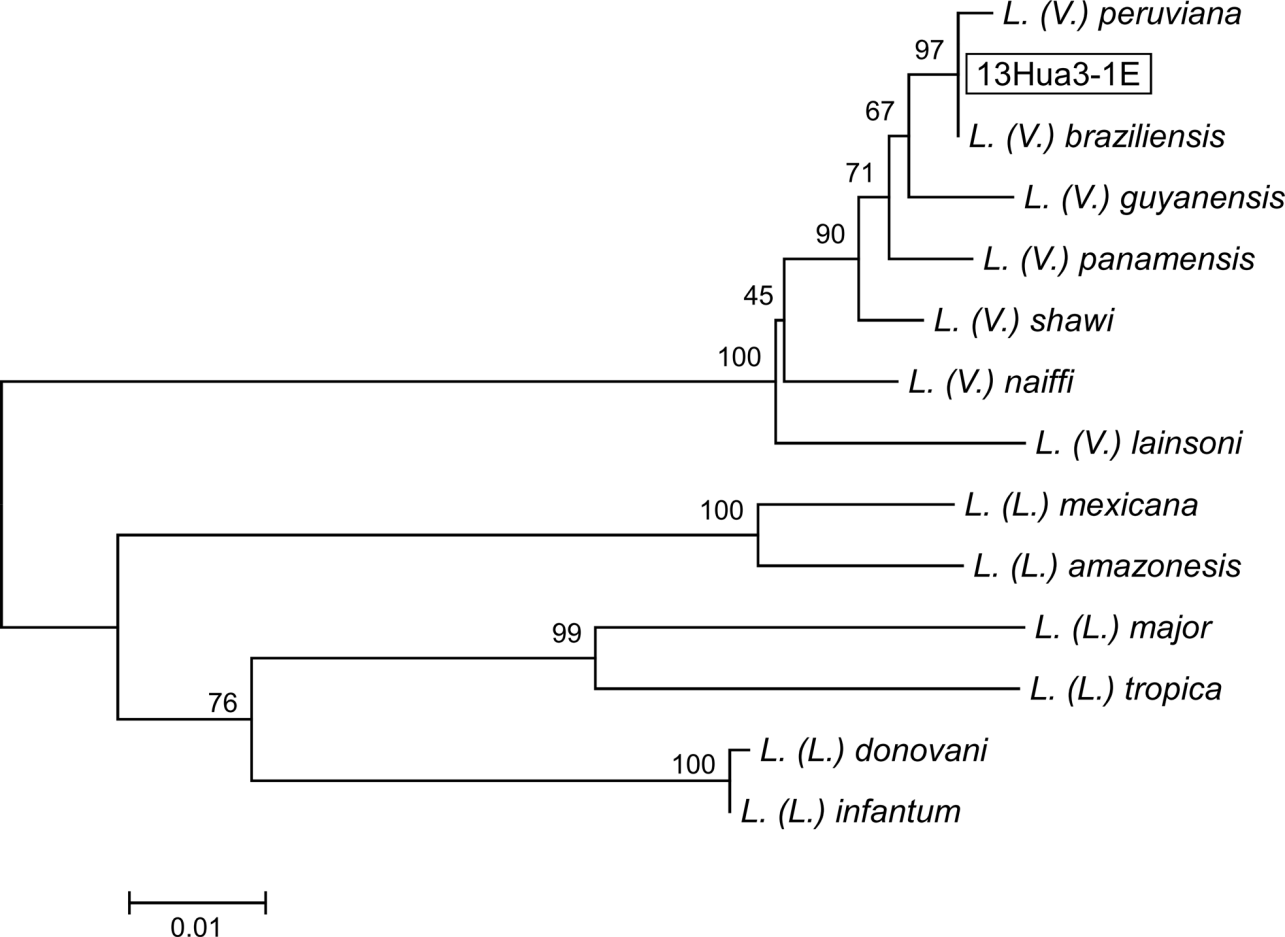
Phylogenetic tree of cytochrome *b* (*cyt* b) gene sequences among species. Leishmanial *cyt* b genes were amplified and sequenced from a sand fly, *Lutzomyia tejadai* (13Hua3-1E). A phylogenetic analysis of *cyt* b gene sequences was performed by the neighbor-joining method together with sequences from 13 *Leishmania* species. The scale bar represents 0.01% divergence. Bootstrap values are shown above branches.

**Fig 2:**
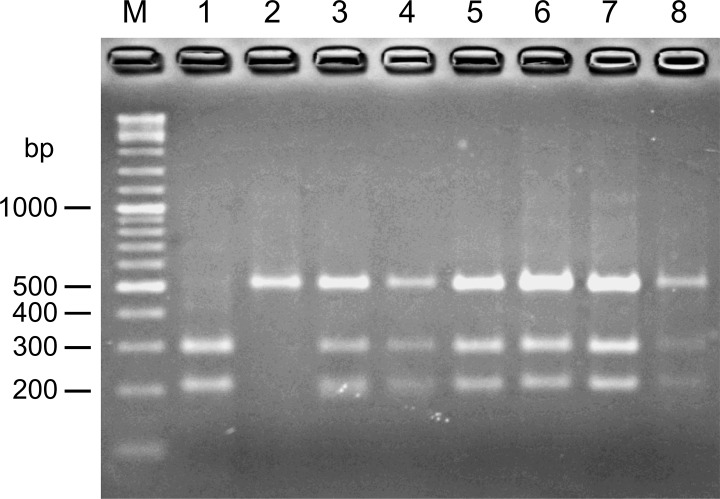
PCR-RFLP analysis of mannose phosphate isomerase (MPI) genes from *L*. *(V*.*) peruviana* (lane 1), *L*. *(V*.*) braziliensis* (lane 2), a hybrid of *L*. *(V*.*) braziliensis/L*. *(V*.*) peruviana* strains LH1099 (lane 3), LC1407 (lane 4), LC1408 (lane 5), LC1418 (lane 6), and LC1419 (lane 7), and *Leishmania*-positive sand fly 13Hua3-1E (lane 8). PCR amplification was performed with MPI gene-specific primers and the PCR products were digested with *Ava*II. Lane M, DNA molecular weight marker.

**Fig 3:**
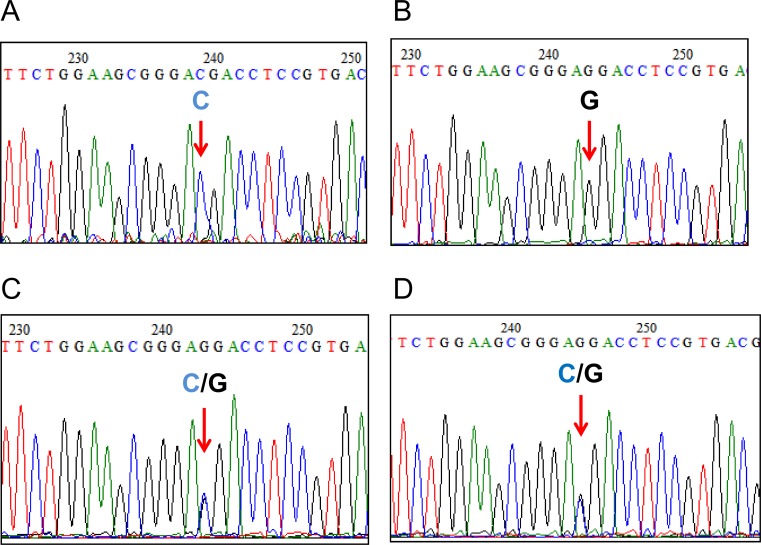
Direct sequence analysis showing a species-specific polymorphic site of *Leishmania* MPI gene fragments. A. *L*. *(V*.*) braziliensis*, B. *L*. *(V*.*) peruviana*, C. a hybrid of *L*. *(V*.*) braziliensis/L*. *(V*.*) peruviana* strain LH1099, D. *Leishmania-*positive *Lu*. *tejadai* 13Hua3-1E.

In this study, only two species of the genus *Lutzomyia*, *Lu*. *tejadai* and *Lu*. *fischeri* were collected. Namely, a total of 2,997 female sand flies were captured and identified at the species level, of which 2,931 and 66 flies were identified as *Lu*. *tejadai* and *Lu fischeri*, respectively. Of these, *Leishmania* minicircle DNA was detected from one *Lu*. *tejadai* from Chinobamba (13Hua3-1E). The sand fly positive for *Leishmania* DNA did not contain blood in the gut. The *cyt* b gene sequence from parasites within the *Lu*. *tejadai* 13Hua3-1E was successfully obtained, and the nucleotide sequence was analyzed. The sequence of parasites from 13Hua3-1E had a greater degree of homology with those of *L*. *(V*.*) braziliensis* and *L*. *(V*.*) peruviana* (99.7–100.0%) than with other *Leishmania* species (88.8–98.8%). The result was supported by a phylogenetic analysis showing that that the specimen from *Lu*. *tejadai* was located in the clade of *L*. *(V*.*) braziliensis* and *L*. *(V*.*) peruviana* (Fig 1). To further identify the species infecting the sand fly *Lu*. *tejadai*, leishmanial MPI gene sequences were analyzed by PCR-RFLP, since a single nucleotide polymorphism of the gene was reported to be a marker for differentiating between *L*. *(V*.*) braziliensis* and *L*. *(V*.*) peruviana* [6,11,19]. As shown in Fig 2, a restriction enzyme, *Ava*II, cut the MPI fragment of *L*. *(V*.*) peruviana* completely, but not that of *L*. *(V*.*) braziliensis*. On the other hand, the MPI fragment of *Leishmania*-positive *Lu*. *tejadai* 13Hua3-1E, as well as those of reference strains of the hybrid (LH1099, LC1407, LC1408, LC1418, and LC1419), showed hybrid patterns after digestion by *Ava*II (Fig 2). The sequences of the MPI fragments were analyzed by direct sequencing, and a single nucleotide polymorphism was confirmed showing “C” in *L*. *(V*.*) braziliensis*, but “G” in *L*. *(V*.*) peruviana* at the corresponding position (Fig 3A and 3B). On the other hand, MPI genes from all reference strains of hybrid (LH1099, LC1407, LC1408, LC1418, and LC1419) and *Leishmania*-positive *Lu*. *tejadai* 13Hua3-1E had both “C” and “G” peaks at the position (Fig 3C and 3D). These results indicated that the parasite species within *Lu*. *tejadai* 13Hua3-1E is a hybrid of *L*. *(V*.*) braziliensis*/*L*. *(V*.*) peruviana*.

## Discussion

Despite their importance in the control of leishmaniasis, little is known about vectors involved in disease transmission since the infection ratio among sand flies by *Leishmania* is generally low (< 1%). CL cases caused by a hybrid of *L*. *(V*.*) braziliensis/L*. *(V*.*) peruviana* have been reported in Peru since 1995; however, the vector species remains unidentified. The present study utilized a molecular mass-screening method for analysis of 2,997 female sand flies from the Department of Huanuco, Peru, in which CL caused by *L*. *(V*.*) braziliensis*, *L*. *(V*.*) peruviana*, *L*. *(V*.*) guyanensis*, and a hybrid of *L*. *(V*.*) braziliensis/L*. *(V*.*) peruviana* is endemic [10,12,20]. As a result, a hybrid of *L*. *(V*.*) braziliensis*/*L*. *(V*.*) peruviana* was detected in one *Lu*. *tejadai* which did not contain host blood in the gut, suggesting that *Lu*. *tejadai* supports the development of the hybrid *Leishmania* and is responsible for its transmission in this area.

In the present collection, only two *Lutzomyia* species were captured, although three species of sand flies, *Lu*. *tejadai*, *Lu fischeri* and *Lu*. *sallesi* have been recorded in the Department of Huanuco [21]. The first species, *Lu*. *tejadai* was collected inside, outside and around houses, suggesting its wide range of distributions and activities in the areas [22]. To date, natural infections of *Lu*. *sallesi* and *Lu*. *fischeri* by *L*. *(L*.*) infantum* and *Leishmania* (*Viannia*) species, respectively, have been reported in Brazil [23,24]. However, infection of these sand flies by *Leishmania* has not been reported in Peru. Furthermore, there is no report on the natural infection of *Lu*. *tejadai* by *Leishmania* species. The present study suggested *Lu*. *tejadai* is the vector of a hybrid of *L*. *(V*.*) braziliensis/L*. *(V*.*) peruviana* for the first time. A hybrid of *Leishmania* caused by genetic exchange is experimentally generated in the digestive tract by co-infecting vector sand fly species with two different strains of the same *Leishmania* species [25–27]. In addition, a direct evidence of sexual recombination in natural population was recently provided by whole genome sequencing of *Leishmania* isolated from sand flies [28]. Midgut molecules of sand fly species are considered to be a major determinant of parasite-vector specificity [29]. Since *L*. *(V*.*) braziliensis* and *L*. *(V*.*) peruviana* are closely-related, it is possible that they share a “*Leishmania* receptor” in the sand fly gut which enables them to develop in *Lu*. *tejadai* and, consequently, hybrids could be generated. Further research into the vector species of *L*. *(V*.*) braziliensis* and *L*. *(V*.*) peruviana* in this area may help to validate this hypothesis. Hybrids of *Leishmania* such as *L*. *(V*.*) braziliensis/L*. *(V*.*) guyanensis*, L. *(L*.*) infantum/L*. *(L*.*) major*, and *L*. *(L*.*) donovani/L*. *(L*.*) aethiopica* have also been reported from other countries, but the vector species remain unidentified [30–32]. Since their parental species transmitted by different vectors were relatively divergent when compared to the relationship between *L*. *(V*.*) braziliensis* and *L*. *(V*.*) peruviana*, the generation mechanism of a hybrid *L*. *(V*.*) braziliensis*/*L*. *(V*.*) peruviana* may be different from those of other hybrids.

The study area, the Department of Huanuco, is located at the mid-eastern region of the Peruvian Andes and is surrounded by seven leishmaniasis-positive departments (San Martin, Ancash, Lima, La Libertad, Loreto, Ucayali, and Pasco). Because of poverty and infertile farm land in the Andean highlands, the inhabitants started to move from the highlands to the lowlands (tropical region) together with their domestic animals (dogs, cats, guinea pigs, cows, sheep, goat, etc.), and some groups migrated to the Department of San Martin, the highest leishmaniasis-endemic area in the country. Around 1975, the number of migrants in these areas increased markedly, and further, in 1983–1984, a massive movement of military personnel from the highlands to the lowlands and *vice versa* occurred in and around the Department of Huanuco for the purpose of narcotic and guerrilla control in the areas (Personal communication: 2013, Hospital Regional Hermilio Valdizan Medrano—Huanuco, DIRESA Huanuco). Such a dynamic and diverse migration of people and animals infected with *Leishmania* parasites in highland or lowland areas may have caused infections by multiple species in humans, reservoir hosts, and sand flies, resulting in the establishment of a hybrid.

The present study detected, for the first time, a hybrid of *Leishmania* in a sand fly. The result suggested that *Lu*. *tejadai* is the responsible vector species of a hybrid *L*. *(V*.*) braziliensis/L*. *(V*.*) peruviana* in the study area in Peru. Since the two parasite species, *L*. *(V*.*) braziliensis* and *L*. *(V*.*) peruviana*, are closely-related, this is a unique natural model for genetic exchange and generation of a hybrid of the genus *Leishmania*.
